# In this issue: Volume 112, Issue 12, December 2021

**DOI:** 10.1111/cas.15200

**Published:** 2021-12-05

## Notch‐1 signaling promotes reattachment of suspended cancer cells by cdc42‐dependent microtentacles formation

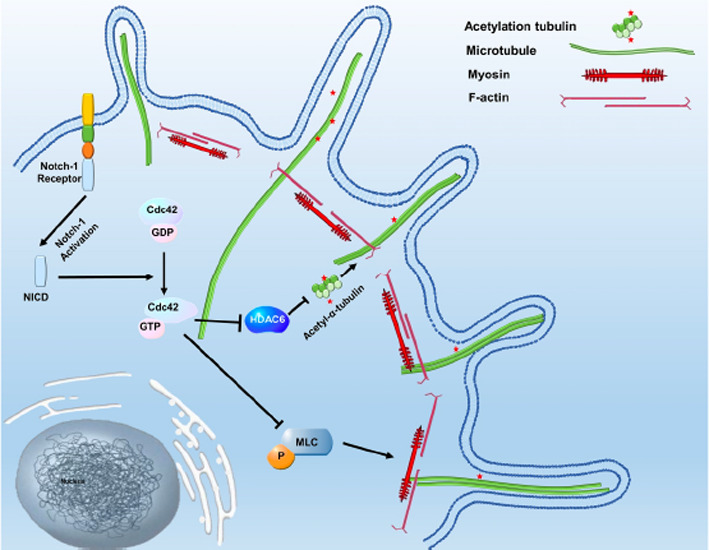

Metastasis is a complex process. There are many steps of the metastatic process that require further study to improve our care of cancer patients with progressive disease. One step of interest is how cancer cells reattach and invade at a new site. Studies have shown that tumor cell reattachment is facilitated by dynamic membrane protrusions called microtentacles (McTNs). In this study, Li et al examine the Notch signaling pathway and the role it plays in the formation of these McTNs. They constitutively activated Notch‐1 in MDA‐MB‐231 and MCF‐7 cells and were able to induce McTNs formation, which resulted in increased cancer cell reattachment. Furthermore, they showed that the McTNs formation was driven by cdc42, a cytoskeletal Rho‐GTPase, activation. Therapeutics that target this mechanism regulating McTNs formation may prevent cancer metastasis. https://onlinelibrary.wiley.com/doi/10.1111/cas.15146

## Crucial contribution of GPR56/ADGRG1, expressed by breast cancer cells, to bone metastasis formation

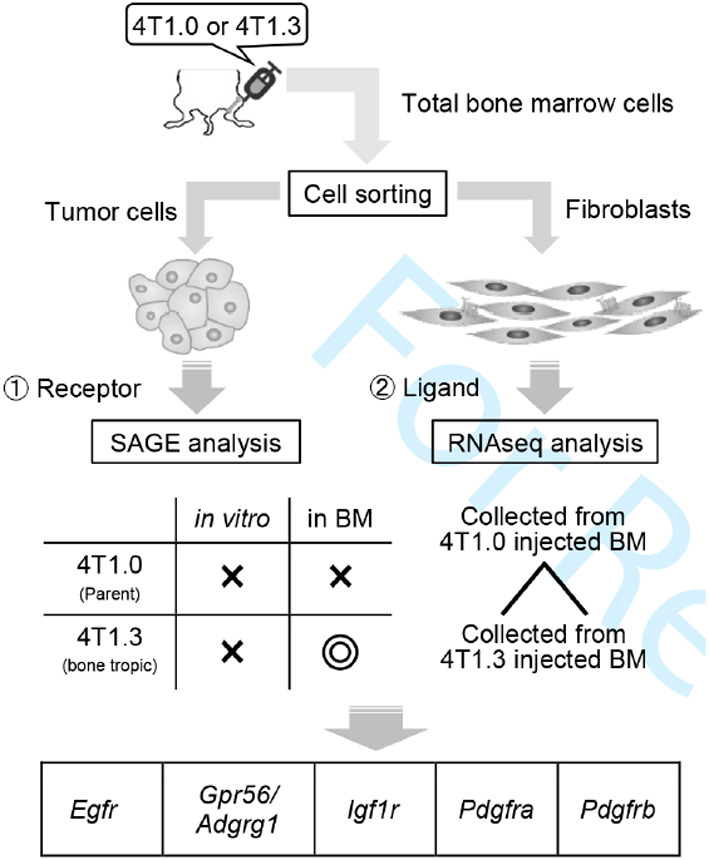

Bone is one of the most common sites of metastasis for breast cancer patients. These bone metastasis are correlated with decreased overall survival and a significant decline in quality of life for these patients. Once in the bone, cancer cells modify the bone marrow microenvironment, consisting of many cell types, including hematopoietic cells, osteoclasts, osteoblasts, mesenchymal stromal cells, and fibroblasts. In this study, Sasaki et al. focused on the interaction between tumors and fibroblasts and identified G‐protein‐coupled receptor 56 (GPR56)/adhesion G‐protein‐coupled receptor G1 (ADGRG1) as the receptor responsible for tumor cell proliferation expressed on tumor cells in bone. They noted that GPR56/ADGRG1 interacts with type III collagen expressed by fibroblasts. This interaction led to reduction in apoptosis and progression of the cell cycle. Targeting specific metastatic tumor microenvironments may be the key to treating bony metastases. https://onlinelibrary.wiley.com/doi/10.1111/cas.15150

## C/EBPΒ induces B‐cell acute lymphoblastic leukemia and cooperates with BLNK mutations

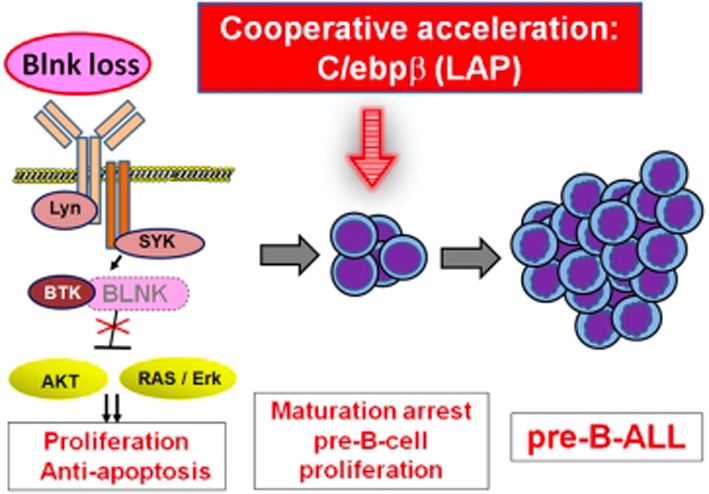

Acute lymphoblastic leukemia is the most common childhood cancer and up to 85 percent of these cases are of B cell origin. Aberrant B‐cell receptor (BCR) signaling is seen frequently in pre‐B‐ALL. Many studies have shown that the downregulation of BLNK is necessary, but not sufficient for leukemogenesis. In this study, Kurata et al used a Moloney murine leukemia virus (MoMLV)‐based retroviral tagging system to identify genes that cooperate with BLNK mutations to promote leukemogenesis. They infected *Blnk* homozygous KO mice with MoMLV and identified *Cebpb* as a common insertion site during the development of pre‐B‐ALL. They found that these mouse models had elevated levels of *Cebpb*‐encoded liver activating protein (LAP), which significantly accelerated the development of pre‐B‐ALL. This mechanism may be targeted to improve the outcomes of childhood cancers. https://onlinelibrary.wiley.com/doi/10.1111/cas.15164

